# No evidence for a decreased risk of thyroid cancer in association with use of metformin or other antidiabetic drugs: a case-control study

**DOI:** 10.1186/s12885-015-1719-6

**Published:** 2015-10-16

**Authors:** Claudia Becker, Susan S. Jick, Christoph R. Meier, Michael Bodmer

**Affiliations:** 1 Basel Pharmacoepidemiology Unit, Division of Clinical Pharmacy and Epidemiology, Department of Pharmaceutical Sciences, University of Basel, St. Johanns-Vorstadt 27, 4031 Basel, Switzerland; 2 Boston Collaborative Drug Surveillance Program, Boston University School of Public Health, Lexington, MA USA; 3 Hospital Pharmacy, University Hospital Basel, Basel, Switzerland

**Keywords:** Antidiabetic drugs, Thyroid cancer, Metformin, Case-control study, Epidemiology

## Abstract

**Background:**

Use of metformin has been associated with a decreased cancer risk. We aimed to explore whether use of metformin or other antidiabetic drugs is associated with a decreased risk for thyroid cancer.

**Methods:**

We conducted a case-control analysis (1995 to 2014) using the U.K.-based Clinical Practice Research Datalink (CPRD). Cases had a first-time diagnosis of thyroid cancer, six controls per case were matched on age, sex, calendar time, general practice, and number of years of active history in the database prior to the index date. We assessed odds ratios (ORs) with 95 % confidence intervals (95 % CI), adjusted for body mass index (BMI), smoking, and diabetes mellitus.

**Results:**

In 1229 cases and 7374 matched controls, the risk of thyroid cancer associated with ever use of metformin yielded an adjusted OR of 1.48, 95 % CI 0.86–2.54. The relative risk estimate was highest in long-term (≥30 prescriptions) users of metformin (adjusted OR 1.83, 95 % CI 0.92–3.65), based on a limited number of 26 exposed cases. No such association was found in users of sulfonylurea, insulin, or thiazolidinediones (TZD). Neither a diabetes diagnosis (adjusted OR 1.17, 95 % CI 0.89–1.54), nor diabetes duration >8 years (adjusted OR 1.22, 95 % CI 0.60–2.51) altered the risk of thyroid cancer.

**Conclusion:**

In our observational study with limited statistical power, neither use of metformin nor of other antidiabetic drugs were associated with a decreased risk of thyroid cancer.

## Background

Thyroid cancer is by far the most common malignant endocrine tumor but accounts for only 2 % of all malignant neoplasms in the U.S. [1]. Thyroid carcinomas are mostly from the differentiated type (95 %) while anaplastic types are rare [2]. Women are three times more often affected than men [2]. The overall incidence of thyroid cancer has risen in recent years both in men and women and across different countries [1, 2]. This increasing incidence is only partially explained by earlier detection of subclinical disease and by increased sensitivity of diagnostic tests, since the incidence of tumors of all sizes has risen in recent years [1–3].

Risk factors for thyroid cancer include exposure to radiation in childhood [4] and rare genetic causes such as a family history of the thyroid cancer syndrome [5]. Data from observational studies suggest that benign thyroid disease [6, 7], hyperthyroidism [8], refraining from smoking and alcohol consumption [9], and high body mass index (BMI) [10] may be associated with an increased risk of thyroid cancer. Diabetes mellitus has not been associated with an altered risk of thyroid cancer in most studies [11].

In recent years, use of the antidiabetic drug metformin has been linked to a decreased risk of some but not all cancer types [12–15]. Furthermore, metformin showed evidence of antitumor activity in various cancer cell lines [16–19] and also in thyroid cancer cell lines [20]. Proposed mechanisms include reduction of mammalian target of rapamycin (mTOR) signalling by activation of adenosine monophosphate activated protein kinase (AMPK), and decreased insulin resistance [21]. The role of AMPK modulation in thyroid tissue has only recently been investigated. Abdulrahman and coworkers reported that activation of AMPK by metformin decreased iodide uptake by a rat follicular thyroid cell-line, while iodide uptake was increased by compound C, an inhibitor of AMPK [22]. Metformin decreased cancer cell growth in various thyroid cancer cell models [20, 23], suppressed self-renewal of cancer stem cells [23], and downregulated AMPK-dependent cell signalling [20].

In contrast, Andrade et al. demonstrated that activation of AMPK increased glucose uptake in rat follicular thyroid PCCL3 cells by upregulation of glucose transporter 1 (GLUT1) [24]. It has repeatedly been shown that GLUT1 is overexpressed in thyroid cancer cells compared to normal thyroid tissue [25, 26] and that this may be an indicator of thyroid cancer progression and aggressiveness [24, 26]. Therefore, AMPK activation by metformin could, in theory, lead to increased glucose uptake and thyroid cancer progression. To our knowledge, two published observational studies have so far explored the risk of thyroid cancer in association with use of metformin [6, 27]. In one of these Taiwan-based studies, Tseng [6] did not find an association between ever use of metformin and thyroid cancer but suggested an increased risk of thyroid cancer for users of sulfonylureas. Data from a recently published investigation by the same author, this time using a different study design, suggested a decreased risk of thyroid cancer in patients with diabetes [27].

The primary aim of this study was to explore whether use of metformin or of other antidiabetic drugs is associated with an altered risk of thyroid cancer.

## Methods

### Data source

Data were derived from the U.K.-based Clinical Practice Research Datalink (CPRD), the former General Practice Research Database (GPRD), a large primary care database which was established in 1987. It encompasses data on some 7 million individuals registered with selected general practitioners (GPs) [28]. Patients enrolled in participating practices are representative of the U.K. with regard to age, sex, and geographic distribution. GPs have been trained to record medical information including demographic data, medical diagnoses, hospitalizations, deaths, and drug prescriptions for research purposes using standard software and standard coding systems. They generate prescriptions directly with the computer, and this information is automatically transcribed into the computer record. The medical record contains the name of the preparation, instructions for use, route of administration, dose, and number of tablets for each prescription. Additionally, the CPRD holds information regarding lifestyle variables such as BMI, smoking, and alcohol consumption, and information on symptoms, medical diagnoses, referrals to consultants, and hospitalizations. The recorded information on drug exposure and diagnoses has repeatedly been validated and has proven to be of high quality [29, 30]. The CPRD has been the source of many observational studies, including research on diabetes and on antidiabetic drugs [31, 32] as well as on cancer [12, 33, 34]. The study was approved by the Independent Scientific Advisory Committee (ISAC) for the Medicines and Healthcare products Regulatory Agency (MHRA) database research.

### Study population

#### Case patients

We used medical READ codes [29] to identify all subjects below the age of 90 years in the CPRD who had a first-time diagnosis of thyroid cancer between January 1995 and December 2014. We excluded all patients with less than 3 years of active history (defined as having been actively registered and having had the opportunity for recordings of either a diagnosis, a drug prescription, an immunization or a lab value in the CPRD database after January 1988) in the database prior to the date of the thyroid cancer diagnosis (subsequently referred to as ‘index date’). Those with a history of any other cancer (except non—melanoma skin cancer), alcoholism (i.e. pathological alcohol consumption), or HIV infection prior to the original index date were also excluded. We shifted the index date for both cases and controls 2 years backward in time for all analyses to reduce the risk of protopathic bias where clinical symptoms of the cancer may have led to modification of the antidiabetic drug treatment and to ensure that exposure to antidiabetic drugs indeed preceded the onset of cancer. Additionally, we assessed whether cases had recorded radiotherapy (including iodine-131 radiotherapy), chemotherapy, thyroid surgery, or specific oncology codes, all indicators of the validity of the cancer diagnosis.

### Control patients

From the base population we identified six controls with no diagnosis of thyroid cancer for each case at random, matched on calendar time (same index date), age (same year of birth), sex, general practice, and number of years of active history on the database prior to the index date. Therefore, the observation period for both cases and controls was the period between the date of entry into the CPRD and the index date. The same exclusion criteria were applied to controls as to cases.

### Exposure to antidiabetic drugs, diabetes mellitus, and diabetes duration

The exposure of interest was the use of different antidiabetic drugs (insulin, metformin, sulfonylurea and thiazolidinediones [TZD]) prior to the shifted index date for cases and controls. In addition to analyzing the effect of any use compared to non-use of the respective antidiabetic drug, we defined several exposure levels based on the recorded number of prescriptions for metformin and sulfonylureas and classified patients into short-term (1–29 prescriptions) or long-term (≥30 prescriptions) users. Since exposure to TZD and insulin was rare, we could not study different prescription categories for these drug classes. We further assessed whether cases and controls had a diagnosis of diabetes mellitus recorded prior to the index date. We also assessed diabetes duration (categorized into the three categories <4 years, 4–8 years, and >8 years), as well as the last recorded level of glycosylated hemoglobin (A1C) prior to the index date.

### Covariates and sensitivity analyses

In addition to diabetes mellitus we assessed the prevalence of various other comorbidities prior to the index date such as hypothyroidism or hyperthyroidism, goiter, cardiovascular diseases (congestive heart failure, ischemic heart disease, stroke or transient ischemic attack, arterial hypertension), and dyslipidemia in cases and controls. Additionally, we classified cases and controls according to their smoking status (non-smoker, current, past, unknown), alcohol consumption (none, current, past, unknown), and BMI (<25 kg/m^2^, 25–29.9 kg/m^2^, >30 kg/m^2^, unknown). Finally, we explored the association between exposure to acetylsalicylic acid (ASA), other non-steroidal anti-inflammatory drugs (NSAIDs), or statins and the risk of thyroid cancer in bivariate analyses as they have been previously associated with an altered risk of thyroid cancer [35–37].

We conducted a sensitivity analysis in patients with diabetes mellitus, i.e. we compared exposure to antidiabetic drugs between cases and controls who all had diagnosed diabetes in order to evaluate potential confounding by indication. For this analysis, we identified additional controls with a diabetes diagnosis (but without a cancer diagnosis) from the database in order to a match one diabetic cancer case to six diabetic controls. We also assessed the influence of diabetes duration and A1C as potential confounders. Since A1C did not materially change our findings in bivariate analyses, we did not include it in the multivariate sensitivity analysis.

### Statistical analysis

We conducted conditional logistic regression analyses using the SAS statistical software version 9.4 (SAS Institute Inc, Cary, NC) to calculate relative risk estimates of insulin use or oral antidiabetic drug use among cases with thyroid cancer, compared with controls without thyroid cancer, expressed as odds ratios (ORs) with 95 % confidence intervals (CIs). We *a priori* adjusted for the potential confounders BMI, smoking, and a recorded diagnosis of diabetes mellitus (or diabetes duration in the sensitivity analysis restricted to diabetic cases and controls) in the multivariate model. We also explored the crude association between predefined covariates as discussed above and the risk of thyroid cancer. Moreover, we assessed the effect of these covariates on the relative risk estimate by including them one by one in our *a priori* model. Since hyperthyroidism and goiter are also known risk factors for thyroid cancer and they yielded high OR in our univariate analyses, we decided to present a second multivariate model including those two variables.

## Results

We identified 1229 cases with an incident diagnosis of thyroid cancer and 7374 matched controls. Mean (± standard deviation [SD]) age at diagnosis was 51.4 ± 17.7 years, and 74.5 % of cases were female. Mean (± SD) recorded history in the database prior to the diagnosis date was 4035 ± 1838 days for cases and 4040 ± 1827 days for controls. Table 1 displays detailed demographic data of cases and controls. Eighty-eight percent of cases had recorded codes for oncologic evaluation, thyroid surgery, radio- or chemotherapy within 6 months before or after the diagnosis date.

**Table 1:** Characteristics of patients with thyroid cancer and their controls

		Cases (%) (*n* = 1229)	Controls (%) (*n* = 7374)	Crude OR (95 % CI)
Age (years)	<40	335 (27.3)	2010 (27.3)	-
	40–59	455 (37.0)	2745 (37.2)	-
	60–69	206 (16.8)	1200 (16.3)	-
	70–79	174 (14.2)	1064 (14.4)	-
	≥80	59 (4.8)	355 (4.8)	-
Sex	Male	314 (25.6)	1884 (25.6)	-
	Female	915 (74.5)	5490 (74.5)	-
BMI	<25	435 (35.4)	2717 (36.9)	1.00 (referent)
	25–29.9	337 (27.4)	1977 (26.8)	1.07 (0.92–1.26)
	30–59.9	245 (19.9)	1222 (16.6)	1.27 (1.07–1.51)
	Unknown	212 (17.3)	1458 (19.8)	0.85 (0.69–1.04)
Smoking	Non-smoker	693 (56.4)	3603 (48.9)	1.00 (referent)
	Current	182 (14.8)	1406 (19.1)	0.66 (0.55–0.79)
	Past	235 (19.1)	1560 (21.2)	0.78 (0.66–0.92)
	Unknown	119 (9.7)	805 (10.9)	0.72 (0.55–0.92)
Alcohol consumption	None	244 (19.9)	1233 (16.7)	1.00 (referent)
	Current	819 (66.6)	4989 (67.7)	0.81 (0.69–0.96)
	Past	13 (1.1)	74 (1.0)	0.88 (0.48–1.62)
	Unknown	153 (12.5)	1078 (14.6)	0.67 (0.52–0.85)
Hypothyroidism	No	1157 (94.1)	7022 (95.2)	1.00 (referent)
	Yes	72 (5.9)	352 (4.8)	1.25 (0.96–1.63)
Hyperthyroidism	No	1195 (97.2)	7284 (98.8)	1.00 (referent)
	Yes	34 (2.8)	90 (1.2)	2.29 (1.54–3.42)
Goiter^a^	No	1129 (91.9)	7313 (99.2)	1.00 (referent)
	Yes	100 (8.1)	61 (0.8)	10.60 (7.62–14.74)
Diabetes mellitus	No	1159 (94.3)	7009 (95.1)	1.00 (referent)
	Yes	70 (5.7)	365 (5.0)	1.17 (0.89–1.54)
	DM duration^b^ < 4 ys.	24 (34.3)	142 (33.9)	1.00 (referent)
	DM duration^b^ 4–8 ys.	18 (25.7)	133 (31.7)	0.71 (0.32–1.60)
	DM duration^b^ > 8 ys.	28 (40.0)	144 (34.4)	1.22 (0.60–2.51)
	A1c <53 mmol/l (7 %)	32 (45.7)	175 (41.8)	1.00 (referent)
	A1c ≥ 53 mmol/l (7 %)	32 (45.7)	217 (51.8)	0.80 (0.46–1.38)
	Unknown A1c level	6 (8.6)	27 (6.4)	1.24 (0.44–3.48)
CHF	No	1217 (99.0)	7283 (98.8)	1.00 (referent)
	Yes	12 (1.0)	91 (1.2)	0.78 (0.42–1.45)
IHD	No	1160 (94.4)	6934 (94.0)	1.00 (referent)
	Yes	69 (5.6)	440 (6.0)	0.93 (0.70–1.23)
Hypertension	No	968 (78.8)	5918 (80.3)	1.00 (referent)
	Yes	261 (21.2)	1456 (19.8)	1.13 (0.95–1.34)
Stroke/TIA	No	1198 (97.5)	7147 (96.9)	1.00 (referent)
	Yes	31 (2.5)	227 (3.1)	0.81 (0.55–1.19)
Dyslipidemia	No	1144 (93.1)	6800 (92.2)	1.00 (referent)
	Yes	85 (6.9)	574 (7.8)	0.86 (0.67–1.11)
NSAID	No prior use	518 (42.2)	3083 (41.8)	1.00 (referent)
	1–4 Rx.	447 (36.4)	2740 (37.2)	0.97 (0.84–1.12)
	≥5 Rx.	264 (21.5)	1551 (21.0)	1.02 (0.85–1.21)
ASA	No prior use	1065 (86.7)	6365 (86.3)	1.00 (referent)
	1–14 Rx.	78 (6.4)	481 (6.5)	0.96 (0.75–1.25)
	≥15 Rx.	86 (7.0)	528 (7.2)	0.96 (0.74–1.26)
Statins	No prior use	1078 (87.7)	6453 (87.5)	1.00 (referent)
	1–14 Rx.	47 (3.8)	337 (4.6)	0.83 (0.60–1.15)
	≥15 Rx.	104 (8.5)	584 (7.9)	1.07 (0.83–1.38)

A1c = glycated hemoglobin, assessed for diabetics only, ‘unknown’ level includes recordings earlier than 1 year before the cancer diagnosis

*OR* odds ratio, *CI* confidence interval, *BMI* body mass index, *CHF* congestive heart failure, *IHD* ischemic heart disease, *TIA* transient ischemic attack, *NSAID* non-steroidal anti-inflammatory drugs, *ASA* acetylsalicylic acid

^a^Goiter includes toxic and nontoxic forms

^b^Analysis restricted to cases and controls with a recorded diagnosis of diabetes mellitus

BMI and cardiovascular comorbidities (including dyslipidemia) were not associated with an altered risk of thyroid cancer (Table 1). Current smoking status (OR 0.66, 95 % CI 0.55–0.79) and current alcohol consumption (OR 0.81, 95 % CI 0.69–0.96) were associated with a decreased risk, while hyperthyroidism (OR 2.29, 95 % CI 1.54–3.42) and goiter (OR 10.60, 95 % CI 7.62–14.74) were associated with increased risks of thyroid cancer. Use of ASA, NSAIDs, and statins had no effect on the risk of thyroid cancer, nor did diabetes mellitus or prolonged diabetes duration (Table 1).

Any prior use of metformin yielded an adjusted OR of 1.48 for the risk of thyroid cancer in the main model and of 1.30 in the sensitivity analysis restricted to diabetic cases and controls, although the results were not statistically significant (Tables 2 and 3). When we stratified our analyses according to exposure duration, we observed a higher risk in both models for long-term metformin use (≥30 prescriptions) compared to non-use, with adjusted ORs of 1.83 (95 % CI 0.92–3.65) in the main model, and 1.48 (95 % CI 0.69–3.18) in the sensitivity analysis restricted to diabetic patients (Tables 2 and 3). To address potential bias of exposure time opportunity (time window bias), we explored whether cases with long-term metformin use (≥30 prescriptions) had longer duration of diabetes mellitus than controls and therefore a higher probability of receiving a prescription. Among all cancer cases with long-term metformin use, 81 % had diabetes duration of more than 4 years and 58 % of more than 8 years. Among controls, 95 % had diabetes duration of more than 4 years and 56 % of more than 8 years.

**Table 2:** Use of antidiabetic drugs and the risk of thyroid cancer

Drugs and No. prescriptions	Cases (%) (*n* = 1229)	Controls (%) (*n* = 7374)	Crude OR (95 % CI)	Adjusted OR (95 % CI) model 1^a^	Adjusted OR (95 % CI) model 2^b^
Metformin					
No prior use	1180 (96.0)	7166 (97.2)	1.00 (referent)	1.00 (referent)	1.00 (referent)
Any use	49 (4.0)	208 (2.8)	1.46 (1.05–2.02)	1.48 (0.86–2.54)	1.56 (0.90–2.71)
1–29	23 (1.9)	116 (1.6)	1.23 (0.78–1.94)	1.31 (0.71–2.40)	1.34 (0.72–2.48)
≥30	26 (2.1)	92 (1.3)	1.75 (1.12–2.72)	1.83 (0.92–3.65)	2.07 (1.03–4.18)
Sulfonylurea					
No prior use	1191 (96.9)	7220 (97.9)	1.00 (referent)	1.00 (referent)	1.00 (referent)
Any use	38 (3.1)	154 (2.1)	1.52 (1.05–2.20)	1.42 (0.82–2.46)	1.41 (0.81–2.48)
1–29	19 (1.6)	77 (1.0)	1.51 (0.91–2.51)	1.45 (0.76–2.77)	1.42 (0.73–2.76)
≥30	19 (1.6)	77 (1.0)	1.54 (0.91–2.59)	1.25 (0.62–2.54)	1.23 (0.60–2.53)
Insulin					
No prior use	1211 (98.5)	7287 (98.8)	1.00 (referent)	1.00 (referent)	1.00 (referent)
Any use	18 (1.5)	87 (1.2)	1.25 (0.75–2.10)	1.14 (0.62–2.09)	1.14 (0.62–2.12)
TZD					
No prior use	1220 (99.3)	7333 (99.4)	1.00 (referent)	1.00 (referent)	1.00 (referent)
Any use	9 (0.7)	41 (0.6)	1.32 (0.64–2.72)	0.82 (0.36–1.84)	0.78 (0.34–1.80)

*OR* odds ratio, *CI* confidence interval

^a^Model 1 adjusted for all antidiabetic drugs in the table, BMI, smoking, and diabetes mellitus

^b^Model 2 adjusted for all antidiabetic drugs in the table, BMI, smoking, diabetes mellitus, hyperthyroidism, and goiter

**Table 3:** Use of antidiabetic drugs and the risk of thyroid cancer restricted to cases and controls with a recorded diagnosis of diabetes mellitus

Drugs and No. prescriptions	Cases (%) (*n* = 70)	Controls (%) (*n* = 419)	Crude OR (95 % CI)	Adjusted OR (95 % CI) model 1^a^	Adjusted OR (95 % CI) model 2^b^
Metformin					
No prior use	23 (32.9)	165 (39.4)	1.00 (referent)	1.00 (referent)	1.00 (referent)
Any use	47 (67.1)	254 (60.6)	1.40 (0.78–2.49)	1.30 (0.68–2.47)	1.42 (0.74–2.73)
1–29	21 (30.0)	129 (30.8)	1.21 (0.61–2.39)	1.18 (0.57–2.45)	1.28 (0.61–2.67)
≥30	26 (37.1)	125 (29.8)	1.60 (0.83–3.09)	1.48 (0.69–3.18)	1.67 (0.76–3.67)
Sulfonylurea					
No prior use	32 (45.7)	231 (55.1)	1.00 (referent)	1.00 (referent)	1.00 (referent)
Any use	38 (54.3)	188 (44.9)	1.48 (0.88–2.48)	1.51 (0.83–2.75)	1.46 (0.78–2.72)
1–29	19 (27.1)	97 (23.2)	1.43 (0.76–2.67)	1.56 (0.79–3.10)	1.52 (0.76–3.07)
≥30	19 (27.1)	91 (21.7)	1.53 (0.81–2.89)	1.41 (0.68–2.94)	1.35 (0.62–2.90)
Insulin					
No prior use	52 (74.3)	349 (83.3)	1.00 (referent)	1.00 (referent)	1.00 (referent)
Any use	18 (25.7)	70 (16.7)	1.83 (0.98–3.42)	1.94 (0.97–3.88)	1.84 (0.90–3.76)
TZD					
No prior use	61 (87.1)	366 (87.4)	1.00 (referent)	1.00 (referent)	1.00 (referent)
Any use	9 (12.9)	53 (12.7)	1.03 (0.45–2.33)	0.80 (0.33–1.92)	0.66 (0.26–2.90)

*OR* odds ratio, *CI* confidence interval

^a^Model 1 adjusted for all antidiabetic drugs in the table, BMI, smoking, and diabetes duration

^b^Model 2 adjusted for all antidiabetic drugs in the table, BMI, smoking, diabetes duration, hyperthyroidism, and goiter

Any use of sulfonylureas was associated with a non-significant increased risk of thyroid cancer in both models (Tables 2 and 3), and did not increase with long-term use (≥30 prescriptions: adjusted OR 1.25, 95 % CI 0.62–2.54). Exposure to insulin or TZD was low and did not produce significant results.

## Discussion

The results of this observational study did not show a decreased risk of thyroid cancer in association with the use of metformin. In the relatively small number of patients with long-term metformin exposure, there was some suggestion of a possible association between metformin and thyroid cancer, although this finding was not statistically significant.

The OR was higher in long-term users of metformin, suggesting (but not proving) a possible association between metformin and thyroid cancer. However, confidence intervals varied considerably due to limited statistical power, and our findings need to be confirmed in other large observational studies. No statistically significantly altered cancer risk was observed in users of sulfonylureas or other antidiabetic drugs. To our knowledge, there have been only two epidemiological investigations published to date exploring the risk of thyroid cancer in association with antidiabetic drug treatment. The author of these studies used a population-based reimbursement database in Taiwan [6, 27]. The findings from the first analysis suggested that use of metformin (based on 37 exposed and 906 non-exposed cancer patients) was not associated with a statistically significantly altered risk of thyroid cancer (adjusted OR 0.70, 95 % CI 0.42–1.16). No data were reported on the effect of exposure duration on the risk of thyroid cancer in this study. Similar to our (statistically non-significant) findings, any use of sulfonylureas (based on 52 exposed and 891 non-exposed cancer patients) was associated with an increased risk of thyroid cancer (adjusted OR 1.88, 95 % CI 1.20–2.95), again with no data on exposure duration. In a subsequent cohort study, Tseng reported a markedly decreased risk of thyroid cancer in association with ever use of metformin (model I, HR 0.683, 95 % CI 0.598–0.780), and a trend towards a lower risk associated with long-term metformin use [27]. However, possible limitations in the study design in the second study are, among others, insufficient assessment of exposure time to metformin, and the assignment of an arbitrary artificial study entry date.

Our results are somewhat surprising given that growing available evidence suggests a possible antitumor effect of metformin or other AMPK activators in thyroid cancer cell lines [38, 39]. However, activation of AMPK has also been associated with increased GLUT1 expression in a rat model of thyroid cells, and increased glucose uptake in thyroid cancer cells has been linked to thyroid cancer progression and aggressiveness [24–26]. In addition, it has recently been demonstrated that AMPK is upregulated in human papillary thyroid cancers [40]. On the other hand, the authors of a retrospective analysis of patients with type 2 diabetes mellitus and differentiated thyroid cancer reported a higher remission rate in users of metformin compared to non-users [41], however, the definition of metformin exposure was not reported in detail in this study. Thus, the association between metformin use and thyroid cancer needs to be further elucidated.

Consistent with the findings by Tseng [6], we also observed a possible decreased risk of thyroid cancer in patients exposed to TZD, however, this finding was not statistically significant and was based on a small number of exposed cases and controls in both studies. It has been shown in an in vivo study that TZD reduce serum DPP-4 activity as a result of reduced DPP-4 secretion and that DDP-4 is expressed in differentiated thyroid cancer cells but not in normal human thyrocytes [42]. Further studies are needed to evaluate the role of TZD in patients with thyroid cancer.

In our study, we additionally explored the association between various covariates and development of thyroid cancer. We did not find an increased risk of thyroid cancer in obese patients. In line with other investigations [9], we also found a decreased risk of thyroid cancer in association with current smoking and current alcohol consumption. We did not find evidence for an increased risk of thyroid cancer in patients with diabetes mellitus compared to non-diabetic patients, regardless of diabetes duration. Similar results have been reported by Shih and colleagues in a recent literature review [11] and by Tseng [6]. Benign thyroid disease, namely goiter, and to a lesser extent hyperthyroidism were associated with increased risks of thyroid cancer in our study, and similar findings have been reported by other authors [6–8]. This result may be partially explained by misclassification of benign thyroid disease as thyroid cancer. Other explanations may be that frequent check-ups of benign thyroid lesions increased the likelihood for detecting thyroid cancer, or that lesions initially judged to be benign turned out to be malignant in follow-up investigations. Of note, inclusion of goiter and hyperthyroidism in another sensitivity analysis (model 2) did not materially change the association between antidiabetic drug use and the risk of thyroid cancer.

Recently, use of statins and NSAIDs have been reported to be associated with markedly decreased risks of thyroid cancer of 45 and 90 %, respectively [6]. We did not find such associations, nor did we find decreased risks with increasing duration of use (Table 1). Despite some encouraging results from mechanistic studies using rosuvastatin [43] and lovastatin [44, 45], our results do not support an overall beneficial effect of statins in preventing thyroid cancer.

There are several limitations in this study. First, the results are based on a limited number of cases and controls despite using a large electronic health records database. Therefore, more data is needed to confirm our findings. Second, we may have missed some thyroid cancer cases, and misclassification of benign thyroid lesions as thyroid cancer cannot be ruled out. However, such misclassification would most likely be equally distributed among different antidiabetic drug groups and would therefore introduce a bias towards the null, and not explain the increased risk found in metformin users. In addition, 88 % of cases had a recorded code for cancer-specific therapeutic interventions after the index date, which makes substantial misclassification unlikely. Finally, in the CPRD cancer diagnoses are recorded with high validity and reasonably high agreement with linked cancer registries [46]. Third, despite assessing the role of numerous potential confounders of the association between thyroid cancer and antidiabetic drug use, we cannot exclude residual confounding by unknown variables. However, conditions known to be risk factors for thyroid cancer for which no detailed information was available in the CPRD (e.g. exposure to radiation in childhood, or a history of a familial thyroid cancer syndrome) should not confound the association of interest since they are not likely linked with exposure to a particular antidiabetic drug class. Fourth, our results are most likely only representative of papillary thyroid cancer, because this type accounts for most thyroid cancers [2]. However, we were not in the position to determine the histological subtype of the cancer cases, so it is possible that metformin exposure does not similarly affect the development of other thyroid cancer cells. Fifth, since higher socioeconomic status (SES) is a surrogate for access to diagnostic procedures and has been associated with increased detection of thyroid cancer [3], SES may also be related to antidiabetic drug use, so we cannot fully exclude confounding by SES. However, we matched cases and controls on general practice and at least partially controlled for SES, since patients from the same neighborhood tend to attend the same general practice.

Our study also has several strengths. We used the CPRD, a longitudinal, well-established, and repeatedly validated primary care database designed for research purposes. In addition to the main analysis we ran a sensitivity analysis in diabetic cases and controls which yielded closely similar findings. We carefully assessed the role of potential confounders on the association of interest. Furthermore, by shifting the original index date 2 years back in time we increased the likelihood that antidiabetic drug exposure preceded cancer development, and we minimized the risk that clinical manifestations of evolving cancer led to changes in antidiabetic drug treatment in cases. Additionally, by excluding all patients with less than 3 years of active history prior to the index date, we reduced the risk of including prevalent rather than incident cancer cases. Finally, time-related biases are most likely not an issue in this study; immortal time bias is ruled out because of risk-set sampling of cases and controls, and bias due to different time of exposure opportunity (time window bias) is also not present.

## Conclusion

Neither use of metformin nor of any other anti-diabetes medication was statistically significantly associated with an altered relative thyroid cancer risk in this population-based observational study.

### Consent statement

There was no consent statement required for this study.

## Abbreviations

A1c: Glycosylated hemoglobin
AMPK: Adenosine monophosphate activated protein kinase
ASA: Acetylsalicylic acid
BMI: Body mass index
CI: Confidence interval
CPRD: Clinical Practice Research Datalink
DPP-4: Dipeptidyl peptidase-4
GLUT1: Glucose transporter 1
GPs: General practicioners
GPRD: General Practice Research Database
HIV: Human immunodeficiency virus
HR: Hazard ratio
ISAC: Independent Scientific Advisory Committee
MHRA: Medicines and Healthcare products Regulatory Agency
mTOR: Mammalian target of rapamycin
NSAIDs: On-steroidal anti-inflammatory drugs
OR: Odds ratio
SD: Standard deviation
SES: Socio economic status
TZD: United Kingdom
US: United States (of America)
